# Regulation of actin catch-slip bonds with a RhoA-formin module

**DOI:** 10.1038/srep35058

**Published:** 2016-10-12

**Authors:** Cho-yin Lee, Jizhong Lou, Kuo-Kuang Wen, Melissa McKane, Suzanne G. Eskin, Peter A. Rubenstein, Shu Chien, Shoichiro Ono, Cheng Zhu, Larry V. McIntire

**Affiliations:** 1 Wallace H Coulter Department of Biomedical Engineering Georgia Institute of Technology and Emory University, Atlanta, GA, USA; 2 Institute for Bioengineering and Biosciences, Georgia Institute of Technology and Emory University, Atlanta, GA, USA; 3 Division of Radiation Oncology, Department of Oncology, National Taiwan University Hospital and National Taiwan University Cancer Center, Taipei, Taiwan; 4 Key Laboratory of RNA Biology, Institute of Biophysics, Chinese Academy of Sciences, Beijing, China; 5 Department of Biochemistry, University of Iowa, Iowa City, IA, USA; 6 Department of Bioengineering and Institute of Engineering in Medicine, University of California at San Diego, La Jolla, CA, USA; 7 Department of Pathology, Emory University, Atlanta, GA, USA.

## Abstract

The dynamic turnover of the actin cytoskeleton is regulated cooperatively by force and biochemical signaling. We previously demonstrated that actin depolymerization under force is governed by catch-slip bonds mediated by force-induced K113:E195 salt-bridges. Yet, the biochemical regulation as well as the functional significance of actin catch bonds has not been elucidated. Using AFM force-clamp experiments, we show that formin controlled by RhoA switches the actin catch-slip bonds to slip-only bonds. SMD simulations reveal that the force does not induce the K113:E195 interaction when formin binds to actin K118 and E117 residues located at the helical segment extending to K113. Actin catch-slip bonds are suppressed by single residue replacements K113E and E195K that interrupt the force-induced K113:E195 interaction; and this suppression is rescued by a K113E/E195K double mutant (E/K) restoring the interaction in the opposite orientation. These results support the biological significance of actin catch bonds, as they corroborate reported observations that RhoA and formin switch force-induced actin cytoskeleton alignment and that either K113E or E195K induces yeast cell growth defects rescued by E/K. Our study demonstrates how the mechano-regulation of actin dynamics is modulated by biochemical signaling molecules, and suggests that actin catch bonds may be important in cell functions.

The dynamic reorganization of the actin cytoskeleton in response to mechanical stress, strain, and substrate rigidity is crucial to the mechanosensitivity, mechanotransduction, and adaptations to the changing mechanical environment of cells. Many observations have revealed that actin dynamics in cells can be modulated by force^1^,^2^,^3^. This dynamic process is also tightly regulated by signaling molecules such as formin controlled by Rho-related GTPase^4^,^5^,^6^.

Formins (formin homology proteins) are mostly Rho-GTPase effector proteins involved in actin polymerization and depolymerization. By stabilizing actin nuclei^7^,^8^,^9^ and continuously binding to F-actin barbed ends^10^,^11^,^12^, the active formin homology domain FH2 accelerates nucleation and reduces the rate of elongation and depolymerization at the barbed end^4^,^5^,^6^,^13^ (Fig. 1a). In mDia1, a mammalian formin reported to localize at the site of actin nucleation^14^ and the tip of growing actin filament in cells^12^, the FH2 domain is auto-inhibited by the interaction between the N-terminal diaphanous inhibitory domain (DID) and the C-terminal diaphanous auto-regulatory domain (DAD). Binding of RhoA to the N-terminus relieves the DAD-DID auto-inhibition^4^,^6^,^9^ (Fig. 1b).

Importantly, mechanical forces and GTPase signaling work cooperatively to control actin dynamics. RhoA and formin have been reported to modulate the force-regulation of intracellular actin dynamics, such as the formation and stabilization of the tension-bearing actin stress fiber and focal adhesion structures^15^,^16^ and the reorganization of these structures induced by externally-applied forces^17^,^18^,^19^. For example, the alignment of intracellular actin stress fibers in response to a unidirectional periodic stretch is modulated by RhoA and mDia1. This alignment is perpendicular to the stretch direction when RhoA and mDia1 are involved, but is switched parallel to the stretch direction if RhoA or mDia1 is compromised^17^. These observations motivated us to investigate the molecular mechanism of force regulation of actin dynamics and the modulation of this biophysical regulation by signaling molecules.

Previously, we demonstrated K113:E195-mediated actin catch-slip bonds as a molecular mechanism of force regulation of actin depolymerization kinetics, using a combination of atomic force microscopy (AFM), steered molecular dynamics (SMD) simulation and yeast actin mutagenesis^20^. We showed that an applied tensile force induces the formation of K113:E195 salt bridges between interacting actin subunits, thereby stabilizing the actin bond and prolonging its lifetime (catch bonds) below a threshold; whereas above the threshold, force shortens bond lifetime (slip bonds). Catch-slip bonds may represent a mechanism for the actin cytoskeleton to contribute to mechano-sensing and mechanotransduction of the cell. In particular, they provide a plausible explanation for the actin-mediated morphological changes of the cell in response to a changing mechanical environment. Examples of changes are the tension-induced assembly and stabilization of the actin cytoskeleton^2^,^21^ and the differential actin stress fiber formation in cells plated on rigid versus soft substrates^3^,^22^.

Assuming the K113:E195 interaction provides, at least in part, a molecular basis for actin catch-slip bonds, the supportive evidence for the potential functional significance of actin catch-slip bonds may come from experiments perturbing the K113:E195 interaction in live cells and disease-causing mutations on either actin K113 or E195 residue. It was shown that interrupting the K113:E195 interaction by either single residue mutation K113E or E195K causes growth defects in yeast cells. These defects included decreased growth, increased cell size, and loss of long polarized actin cytoskeletons; and they are rescued by E/K double mutants that combines both K113E and E195K thus restoring the K:E interaction in the opposite orientation^23^. In addition, in a helical segment spanning between K113 at the inter-strand interface of contacting actin monomers and K118 on the actin filament surface^24^, virtually every residue is mutated in a disease^25^, with the K113E mutation in human α-actin reported as a cause for nemaline myopathy^26^. These observations further support the functional importance of the K113:E195 interaction in cells.

Here we examine factors proven to perturb actin-related cellular phenotypes, for their potential effects on K113:E195-mediated actin catch-slip bonds. We determine whether and how actin catch-slip bonds are modulated by RhoA and formin that regulates the force-controlled cytoskeletal dynamics^17^. Our previous study showed that neutral mutations K113S and E195S, which potentially destroy the K113:E195 ionic bridge, eliminate actin catch-slip bonds. Therefore, we also test the effect of oppositely charged mutations K113E, E195K and E/K, which were shown to perturb cell functions in yeasts^23^ and display a pathological phenotype^26^.

## Results

### Formin switches actin catch-slip bonds to slip-only bonds

To investigate whether and how formin modulates the mechanical regulation of actin dissociation kinetics, we performed AFM force-clamp experiments^20^. In these experiments, a cantilever tip bearing G-actin was allowed to interact with G-actin or F-actin coated on a polystyrene surface under a constant force (Fig. S1). Control experiments were performed to confirm that the bond measured was specific to actin/actin interaction, that the lifetimes were determined by actin/actin bond but not biotin/strepavidin bond, and that the dissociation of G-actin/F-actin bond was at the end but not in the middle or the side of actin filament^20^. The buffer and the experimental protocol were designed to minimize the contamination of F-actin fragments at AFM probes functionalized with G-actin, although the possible presence of actin species other than G-actin could not be completely ruled out. At optimal forces where actin catch-slip bonds reach maximum lifetimes (10 and pN for G-actin/G-actin and G-actin/F-actin interactions, respectively^20^), nonspecific interactions occurred much less frequent (Fig. S2a), ruptured more readily to yield smaller fractions (6 and 1% for G-actin/G-actin and G-actin/F-actin, respectively) of interactions that survived the force ramping phase to allow lifetime measurement (Fig. S2b), and lasted significantly shorter (Fig. S2c).

**Figure 1:**
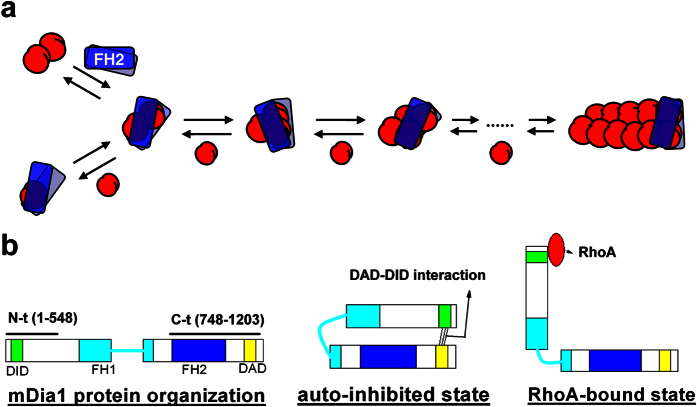
Formin function and regulation. (**a**) The FH2 domain of formin stabilizes actin nuclei (red) and continuously binds to the barbed end of actin filaments while allowing the addition of new actin monomer to the barbed end (modified from cited ref. 8). (**b**) Organization and regulation of mDia1 protein domains. Lines above the left-most diagram represent the constructs used in this study: mDia1 N-t and mDia1 C-t, with the starting and ending amino acids specified. The active FH2 domain is auto-inhibited by the DAD-DID interaction, which is relieved when RhoA binds to the N-terminus to compete with DAD (modified from cited ref. 4).

Here we measured the force-dependent lifetimes of actin subunit interactions in the presence of the FH2 and DAD domain-containing C-terminal construct (C-t) or the DID domain-containing N-terminal construct (N-t) of mDia1 or both constructs with or without RhoA in the working buffer (Fig. 1b). The working buffer of our AFM assay did not change the reported behavior of the formin constructs and His-RhoA on actin kinetics or RhoA-mediated formin inhibition^9^,^27^, which was confirmed by the results of a pyrene actin polymerization assay (Fig. S3). Lifetimes measured in the presence of mDia1 C-t were predominantly mediated by specific G-actin/G-actin or G-actin/F-actin interactions, as the binding frequency was diminished in conditions that prevented these interactions: by coating the cantilever tip with BSA instead of G-actin, by using G-buffer instead of F-buffer as the working buffer, or by adding 2 μM latrunculin A (Fig. S4).

**Figure 2:**
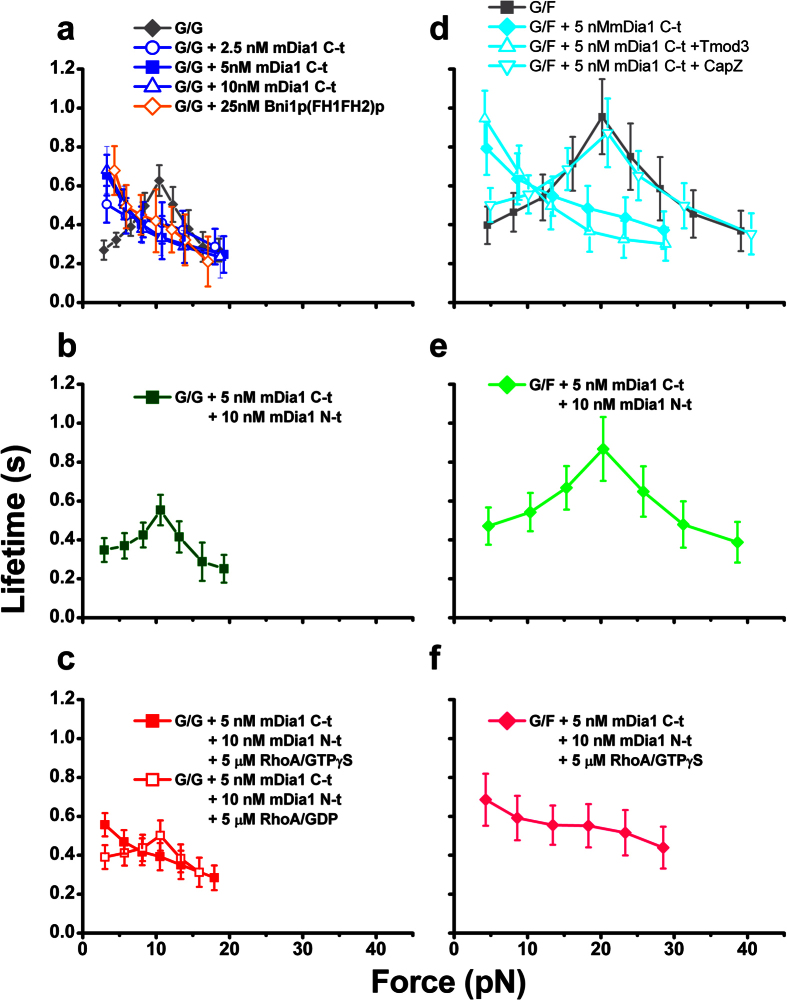
Switching between actin catch-slip bonds and slip bonds by a RhoA-mediated auto-inhibitory module of mDia1. (**a**) mDia1 C-t (blue symbols) or Bni1 (FH1FH2)p (orange open diamond) converted G-actin/G-actin catch bonds (gray diamond, data presented in cited ref. 20) to slip-only bonds, in the force range measured (**b**) mDia1 N-t inhibited the mDia1 C-t-induced conversion of G-actin/G-actin catch-slip bonds to slip-only bonds, restoring the catch-slip phenotype. (**c**) His RhoA charged with GTPγS (red square), but not GDP (red open triangles), relieved the inhibitory effect of mDia1 N-t on mDia1 C-t. (**d**) G-actin/F-actin catch-slip bonds (gray square, data presented in cited ref. 20) were converted to slip-only bonds by 5 nM mDia1 C-t (blue diamond), the effect of which was exacerbated by 2 μM Tmod3 (open up triangle) but diminished by 10 nM CapZ (open down triangle). (**e**) mDia1 C-t-induced conversion from G-actin/F-actin catch-slip bonds to slip-only bonds was inhibited by simultaneous treatment with mDia1 N-t. (**f**) The inhibitory effect mDia1 N-t on mDia1 C-t was relieved by His RhoA charged with GTPγS. Each point represents the mean ± 95% confidence interval (C.I.) of >30 measurements. The confidence interval is calculated as the standard error multiplied by the *t* statistic from the *t* table, assuming a *t*-distribution with a degree of freedom of sample size minus 1. The semilog plots of survival frequency versus lifetime for the G-actin/G-actin interaction with 5 nM mDia1 C-t, G-actin/F-actin interaction with 5 nM mDia1 C-t and 2 μM Tmod3 were shown in the Fig. S9.

The FH2 domain-containing mDia1 C-t facilitates actin nucleation and slows actin depolymerization^9^,^27^. Remarkably, adding mDia1 C-t to the working buffer of the AFM assay switched the G-actin/G-actin catch-slip bonds to slip-only bonds in the force range tested (>3 pN) in a dose-responsive manner (Fig. 2a). A similar effect was caused by Bni1p(FH1FH2)p (Fig. 2a), an FH1 and FH2 domain-containing yeast formin construct which participates in actin nucleation and assembly of the actin filament barbed end^28^,^29^.

In full-length mDia1, the activity of the FH2 domain is auto-inhibited by the DAD-DID interaction (Fig. 1b), and the inhibition remains effective even when the DAD and DID domains are on separate mDia1 C- and N-terminal constructs^9^,^27^. To investigate how this auto-inhibitory mechanism modulates the force regulation of G-actin/G-actin dissociation kinetics, mDia1 N-t was added to the mDia1 C-t-containing AFM assay system. Interestingly, the lifetime vs. force curve of the G-actin/G-actin bonds measured in the presence of both mDia1 C-t and N-t constructs was indistinguishable from that measured in their absence (Fig. 2b). In other words, mDia1 N-t reversed the mDia1 C-t-induced switch of G-actin/G-actin catch-slip bonds to slip-only bonds back to the original catch-slip bonds.

The mDia1 activity is regulated by RhoA, which competitively blocks the DAD-DID interaction and rescues the activity of the auto-inhibited FH2 domain of mDia1^9^,^27^ (Fig. 1b). Indeed, adding His-RhoA loaded with GTPγS to our assay system containing mDia1 C-t and mDia1 N-t relieved the auto-inhibitory effect of the latter on the former, and switched the G-actin/G-actin catch-slip bonds to slip bonds as did mDia1 C-t alone without mDia1 N-t and RhoA (Fig. 2c). Adding GDP-loaded His-RhoA suppressed the catch bonds by shortening their lifetimes at low forces (<10 pN), but did not switch them back to slip bonds, indicating a less potent relieving effect on mDia1 N-t of RhoA loaded with GDP than GTPγS (Fig. 2c).

For the G-actin/F-actin interaction, those constructs of the RhoA-formin module caused the same modulation effects on the force regulation of dissociation kinetics as they did on the G-actin/G-actin dissociation: mDia1 C-t switched the G-actin/F-actin catch-slip bonds to slip-only bonds (Fig. 2d); mDia1 N-t reversed the switch by auto-inhibiting mDia1 C-t (Fig. 2e); and RhoA blocked the auto-inhibition of mDia1 C-t by mDia1 N-t to restore the modulation effect of the FH2 domain, switching the G-actin/F-actin catch-slip bonds to slip-only bonds again (Fig. 2f). The catch-slip to slip-only conversion by mDia1 C-t in G-actin/F-actin interaction was more prominent in the presence of Tmod3 (Fig. 2d), which blocks actin turnover at the pointed end of the filament^20^,^30^. The conversion was diminished by CapZ (Fig. 2d), which blocks the F-actin barbed end^20^,^31^. These results indicate that mDia1 C-t modulates the force-dependent dissociation of G-actin from via the F-actin barbed end but not pointed end, which is consistent with the report that mDia1 associates with the barbed-end of actin filament and this association is inhibited by CapZ^31^.

His-RhoA and mDia1 N-t together had no effect on G-actin/G-actin and G-actin/F-actin catch-slip bonds (Fig. S5). Therefore, the relieving effect of RhoA was specifically on rescuing the activity of mDia1 C-t from mDia1 N-t/mDia1 C-t auto-inhibition.

### SMD-simulated formin-bound actin oligomer under tensile force

**Figure 3:**
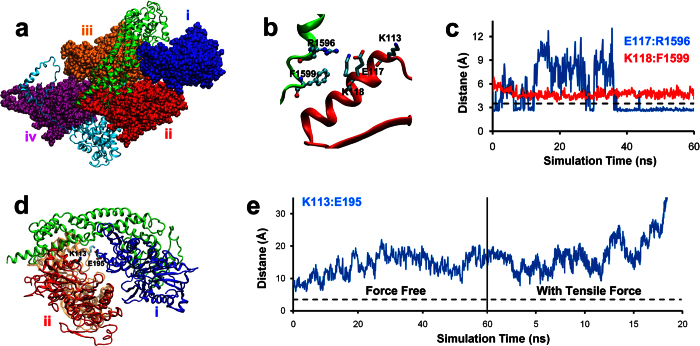
SMD simulations of a structural model of actin barded end oligomer in complex with formin. (**a**) The formin-actin complex model constructed according to the crystal structure 1Y64^11^. Four actin subunits were represented in blue (i), red (ii), orange (iii) and purple (iv), sequentially from bared to pointed end, respectively. Two formin FH2 subunits were displayed as green and cyan. (**b**) The contact interface between formin and the actin helical segment spanning through residues 113 to 118. (**c**) Time courses of distance between the indicated atoms of residues indentified in (**b**). An inter-atomic distance below the 3.5 Å threshold indicates the formation of the salt bridge. (**d**) The FH2 domain-bound inter-strand actin subunits (i and ii) at the barbed end. The barbed-end subunits from the previously-simulated formin-free F-actin^20^ was taken as the reference conformation, with the subunit (i) superimposed (blue) and adjacent subunit (ii) depicted in red (formin-bound) or light orange (formin-free). (**e**) Time courses of distance between K113 and E195. (**c,e**) are representatives of more than 3 simulations for each panel.

To elucidate the structural mechanism for the formin regulation of actin catch-slip bonds, we used SMD simulations to study the atomic-level interactions in a structural model containing two yeast Bni1p FH2 domains in complex with four actin subunits (Fig. 3a)^11^.

In the model, the actin subunit contacts the formin FH2 via its helical segment with K113 on one end at the inter-strand interface and K118 on the other end near the filament surface (Fig. 3b). Under MD simulations, a salt bridge between actin E117 and formin R1596 was observed (Fig. 3c, cyan curve). The distance between actin K118 and formin F1599 remained nearly constant (~4.5 Å), suggesting the formation of a cation-π interaction between the two residues (Fig. 3c, red curve). Actin K118 has also been seen to form contact with formin in another structure that contains mouse formin FMNL3 FH2 domains in complex actin oligomers (PDB 4EAH)^32^.

In the formin-bound structure, the distance between the two actin monomers of the inter-strand dimer at the barbed end is increased (Fig. 3d), compared to that of the previously-simulated barbed-end subunits of the formin-free F-actin^20^. This deformation in the actin dimer induced by formin FH2 binding further separates K113 from the cross-strand opposing E195 residue. This may explain why our previously observed force-induced K113:E195 interaction in the SMD simulations of F-actin without formin^20^ was not observed when tensile force was applied across the formin-bound actin filament in the present SMD simulations (Fig. 3e).

Together, our structural analysis and SMD simulation suggest that formin binds to the surface residues of an actin helical segment terminating at the K113 residue, which increases the distance between barbed end inter-stand subunits, therefore eliminating the K113:E195-mediated actin-catch bond.

### Yeast actin point mutations K113E and E195K suppress actin catch-slip bonds which is rescued by the double mutant E/K

Yeast actin mutagenesis has been applied in combination with the AFM force-clamp experiment to access the importance of the K113:E195 interaction in determining force-dependent lifetimes of actin subunit interactions^20^. Here we use yeast actin as a model system to test the effect of biologically-relevant mutations K113E, E195K and E/K^23^ on actin catch-slip bonds.

**Figure 4:**
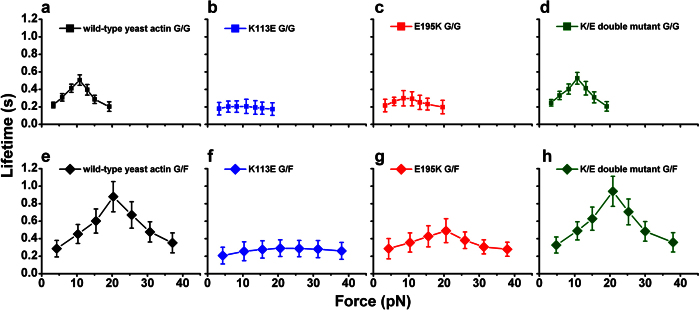
The suppressive effect of K113E and E195K mutations on actin catch-slip bonds was rescued by the E/K double mutant. (**a**) Catch-slip bonds between wild-type yeast actin monomers (data presented in cited reference^20^). (**b,c**) G-actin/G-actin catch-slip bonds were suppressed by either mutation K113E (**b**) or E195K (**c**). (**d**) E/K double mutants restored G-actin/G-actin catch-slip bonds. (**e**) Catch-slip bonds between wild-type yeast actin monomer and filament. (**f**–**h**) G-actin/F-actin catch-slip bonds were suppressed by K113E (**f**) or E195K (**g**), and rescued by E/K double mutants (**h**). Each point represents the mean ± 95% C.I. of >30 measurements. The C.I. is calculated with the same method as described in Fig. 2. The semilog plots of survival frequency versus lifetime were shown in the Fig. S10.

The interaction between two wild-type yeast actin(WYA) monomers has a catch-slip phenotype similar to that between muscle actin monomer and either muscle actin monomer or WYA monomer (Fig. 4a), consistent with our previous results^20^. This finding justifies the extrapolation of results obtained from experiments with the genetically mutatable yeast actin to other systems such as muscle actin, regarding the study of G-actin/G-actin catch-slip bonds.

The G-actin/G-actin catch-slip bonds were suppressed by yeast actin mutant K113E (Fig. 4b) or E195K (Fig. 4c). The suppressive effect of K113E or E195K was reversed by the E/K double mutant (Fig. 4d), which combines both K113E and E195K on a G-actin subunit and therefore may restore the interaction between K and E residues in the opposite orientation (i.e. E113:K195 interaction). These results suggest a physiological role of K113:E195-mediated actin catch bonds in maintaining normal yeast growth, because either K113E or E195K mutation caused growth defects in yeast cells, presumably by interrupting the K113:E195 interaction. Furthermore, the E/K double mutant rescued yeast cell growth defects caused by K113E or E195K, presumably by restoring the K113:E195 interaction^23^.

The catch-slip bonds of G-actin/F-actin interactions in WYA were similar to those observed in muscle actin (Fig. 4e), consistent with our previous results^20^, again justifying the application of the yeast actin model to the study of G-actin/F-actin catch-slip bonds. The G-actin/F-actin catch-slip bonds were suppressed by K113E (Fig. 4f) or E195K (Fig. 4g) mutations on the composing actin subunits. E/K double mutant reversed the suppressive effect of K113E or E195K on G-actin/F-actin catch-slip bonds (Fig. 4h). These results further support the physiological role of the K113:E195-mediated actin catch bonds proposed in the preceding paragraph.

## Discussion

**Figure 5:**
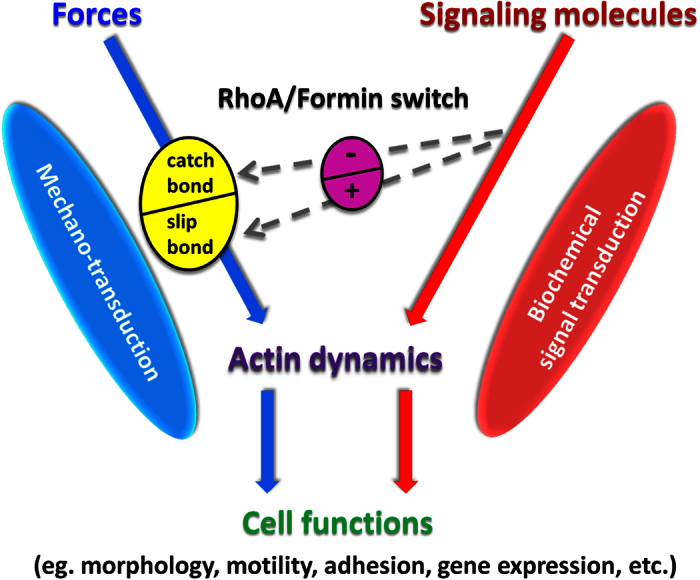
Schematic summary of major concepts. Actin cytoskeleton dynamics are regulated by forces (blue arrows). They are also regulated by GTPases, contributing an important framework for biochemical signal transduction (red arrows). Force regulates actin dynamics by a catch-slip mechanism (yellow circle); and this biophysical regulation is modulated by biochemical signaling through RhoA and formin (gray dashed arrows). RhoA-formin module can serve as a switch shifting the force dependence of actin dynamics between catch bond (with inactivated RhoA or formin) and slip bond (with activated RhoA and formin). It may contribute a crosstalk between mechanotransduction and signal transduction pathways, which control various cell functions.

Actin filaments are the major force-bearing structure in the cytoskeleton. Their dynamic turnover in cells is controlled by forces (Fig. 5, blue) as well as biochemical signaling molecules (Fig. 5, red); and there is substantial evidence suggesting there is cooperative crosstalk between these two regulatory mechanisms^15^,^16^,^17^,^18^. The molecular basis for this biochemical regulation primarily includes GTPase-mediated signaling molecules^4^,^5^,^6^, which have been more established than that of the force regulation^1^,^2^,^3^. We reported actin catch-slip bonds with tensile force-prolonged bond lifetimes for actin subunits interactions caused by tension-induced K113:E195 salt bridges^20^. Actin catch bonds may play a role in the mechanosensing of the cell^3^,^33^ and explain cell functions mediated by the tension-induced assembly and stabilization of the actin cytoskeleton^2^,^3^,^15^,^21^,^22^,^34^, thus providing a plausible molecular basis for the force-regulation of actin dynamics (Fig. 5, yellow). These observations then begged the question as to whether the biophysical and biochemical regulatory mechanisms for actin dynamics are independent or cooperative at the molecular level, and if cooperative, how one molecular mechanism modulates the other.

The first goal of the present paper was to investigate the possible connection and interplay of actin catch bonds and the RhoA-formin module (Fig. 5, purple). These GTPase-mediated signaling molecules were chosen because tension-mediated formation and turnover of the actin cytoskeleton are altered by perturbing formin and RhoA in cells^15^,^16^,^17^,^18^. At the molecular level *in vitro*, formin under stress was shown to affect the elongation rate of the bound actin filament^35^, but the role of formin on the force regulation of actin bond lifetimes was first presented herein. Our finding that these important signaling molecules convert actin catch-slip bonds to slip-only (Fig. 2) attests to the significance of actin catch-slip bonds in cell physiology, and suggests a molecular mechanism for the crosstalk between the biophysical and biochemical regulatory mechanisms (Fig. 5, gray dashed arrows). With formin, tensile force decreases actin bond lifetimes monotonically (slip-only bonds) (Fig. 2), consistent with reported *in-vitro* observations that tension slows formin-mediated actin elongation in the absence of profilin^36^.

At low forces (and extrapolation to zero force), FH2 domain-containing formin constructs mDia1 C-t and Bni1p(FH1FH2)p prolonged actin bond lifetimes (Fig. 2a,d). This is consistent with the previous report that the FH2 domain stabilizes actin dimers and slows F-actin depolymerization at the barbed-end under force-free conditions^7^,^8^,^9^,^13^,^29^. This result could be structurally explained by a model that formin forms an elastic spring-like ring, thus enhancing the stability of bound actin subunits^11^,^37^.

At higher forces, the FH2 domain-containing formin constructs eliminated the lifetime peak of actin catch-slip bonds (Fig. 2a,d). This observation might be explained by our structural model. Central to this model is the actin pathogenic helix, a helical element beginning at actin residue K113 at the inter-strand interface and terminating at the residue K118 on the surface of the actin filament. Virtually every residue in this helix has disease causing mutations^25^. Formin may interact with actin K118 and K117 on the filament surface (Fig. 3a,b)^11^,^32^. This formin-actin interaction at the filament surface is therefore hypothesized to propagate inward through the helix to allosterically interfere with the K113:E195 cross-strand interaction, eliminating the catch bond. This model was supported by our SMD simulation (Fig. 3d,e), showing that K113 and E195 at the barbed-end of formin-bound actin tetramer are too far apart from each other to form the force-induced ionic bridge observed in formin-free actin subunits^20^. This formin-coupled allosteric regulation through the pathogenic helix was further supported by experiments from both bulk solution assay (Fig. S6)^23^ and force-lifetime assay (Figs S7 and S8), showing mutations on K113 perturb the effect of formin on actin kinetics, assuming a retro-propagated conformational change caused by these mutations.

For a cell to function properly, its cytoskeleton must be maintained stably to provide the integrity and form as well as allow dynamic turnover to enable motility and shape changes. Actin catch-slip bonds and their conversion to slip-only bonds by formin could fulfill these seemingly competing requirements for regulating the actin cytoskeleton. At low forces, a formin-free actin filament is more dynamic than a formin-bound actin filament, as the former is shorter-lived than the latter. At higher forces, the stability order is reversed. The formin-free actin filament becomes more stable than the formin-bound actin filament, as force prolongs the lifetime of the former by catch bonds but not the latter because formin eliminated catch bonds (Fig. 2). Thus, formin-regulated actin catch-slip bonds allow the co-existence of both stable and dynamic pools of actin filaments as well as switching of the partitioning of these pools in response to biochemical and mechanical cues.

In G-actin/F-actin interactions, the formin-modulated switch from catch-slip bonds to slip-only bonds shifts the force that corresponds to the maximal bond lifetime from 20 pN to nearly zero (Fig. 2d). This switchable force dependence can generate anisotropic variations in bond lifetimes of actin structures along different directions, thereby resulting in anisotropic stability of the actin network in cells. This may lead to directional alignment of the actin cytoskeleton in cells sustaining anisotropic forces, depending on the activity of RhoA and mDia1. For example, it was observed that bovine aortic endothelial cells and their actin stress fibers align perpendicular to the cyclic uniaxial stretch direction when RhoA and mDia1 are involved, and the alignment is parallel to the stretch direction if RhoA or mDia1 is compromised^17^. These observations imply that with mDia1 and RhoA, the actin cytoskeleton is most stable in the direction of minimal force (perpendicular to stretch); and the direction with the highest stability for the actin cytoskeleton will be shifted to that of maximal force (parallel to stretch) if mDia1 or RhoA is suppressed. This switch might be explained by our findings that the actin dissociation kinetics depend on force in a fashion that involves switching between catch-slip and slip-only bonds controlled by the RhoA-mDia1 pathway.

The second goal of this study was to examine the effect of actin mutants K113E, E195K and E/K on actin catch-slip bonds. These specific mutants were chosen because of their proven functional roles in yeast cells–either K113E or E195K, that potentially interrupts the K113:E195 interaction, and contributes to the yeast cell growth defects including decreased growth, increased cell size, and loss of long polarized actin cytoskeletons; and E/K double mutants that restores the K:E interaction in the opposite orientation rescues these defective phenotypes^23^. As there is no correlated effect of K113E, E195K and E/K in parallel on the force-free bulk actin polymerization assay^23^, effects of these three mutations on yeast cells cannot be consistently explained by their role on the hypothetical K113:E195 interaction in the un-stressed actin filament. Our finding that actin catch-slip bonds are suppressed by either K113E or E195K but restored by E/K (Fig. 4) correlates with the observations in yeast cell phenotypes. Therefore the K113:E195 interaction induced by tensile force (i.e. actin catch-slip bonds) is more likely to be the crucial factor whose perturbation by K113E or E195K contributes to the growth defects in yeast cells.

A crucial question raised by our report of the K113:E195 actin catch-slip bonds^20^ has been the functional significance of this biophysical regulatory mechanism in live cells. We demonstrated the biophysical effects of RhoA-formin module (Fig. 2) as well as K113E, E195K and E/K mutants (Fig. 4) on actin catch-slip bonds, which correlate with the biological effects of these biochemical signaling molecule and actin mutations in the live cells^17^,^23^. In addition, the K113E mutation on human actin gene ACTA1 was reported to be involved in nemaline myopathy^26^. The pathogenic helix with clusters of disease-causing mutations starting at residue K113 further implies the potential pathological significance of the force-induced K113:E195 interaction. Though the direct observation of actin catch-slip bonds in cells is yet not technically feasible, these results on the perturbation of actin catch-slip bonds in parallel to the *in-vivo* observations together provide significant evidence supporting the biological importance of actin catch-slip bonds (Table 1).

In summary, as the first to investigate how the force regulation of actin dynamics is modulated biochemically, our study suggests that the RhoA-regulated DAD-DID auto-inhibitory module of mDia1 functions as a “molecular switch” to modulate the force dependency of actin dynamics. It thus provides a possible mechanism for integrating biophysical and biochemical signaling pathways to control actin cytoskeleton dynamics (Fig. 5).

## Methods

### Proteins

Rabbit skeletal muscle G-actin biotinylated at random surface lysine residues with ~1 biotin per actin monomer was from Cytoskeleton (Denver, CO). Latrunculin A was from Sigma Aldrich (St. Louis, MO).

Generation, purification and biotinylating of WT and mutant yeast actins have previously been described^38^. A detailed protocol is provided in the Supplemental Methods.

Chicken CapZ was expressed in *E. coli* and purified as described^39^. Budding yeast formin construct Bni1p(FH1FH2)p containing FH1 and FH2 domains was a gift from Dr. David Kovar (University of Chicago)^28^. Mouse formin mDia1 constructs (N-terminal: 1–548, C-terminal: 748–1203) in pGex-KT, provided by Dr. Henry N. Higgs (Dartmouth Medical School) were expressed and purified as described previously^9^,^27^. A human tropomodulin Tmod3 construct in pGex-KG, provided by Dr. Velia M. Fowler (Scripps Research Institute) was used to obtain purified Tmod3 as described previously^30^. Concentrations of purified mDia1 constructs and Tmod3 were determined by densitometry of Coomassie blue-stained gels after SDS-PAGE using known amounts of actin as standards. mDia1 proteins were stored at 5 μM at −20 °C with 50% glycerol to avoid loss of activity upon freezing^27^.

His-RhoA (Cytoskeleton) was charged with GTPγS (Cytoskeleton) or GDP (Roche, Nutley, NJ) as described previously^15^. The mixture was incubated at room temperature for 15 min, supplemented with MgCl_2_ to a final concentration of 10 mM, then kept on ice and used within 2 hr.

### AFM force-clamp experiments

Our custom-made AFM and force-clamped experimental procedures for measuring lifetimes of single bonds have been previously described^40^. The protocol and parameters set specifically for the force-dependent actin kinetic assay in this study have been described^20^. A detailed protocol is provided in the Supplemental Methods.

### SMD simulations

The modeled structure of the formin/actin complex containing four actin subunits and two formin FH2 domains was constructed from the crystal structure of the yeast Bni1p formin FH2 domain in complex with rabbit actins (PDB code 1Y64)^11^. The constructed model was equilibrated and the resulting final structure was used for SMD simulation. To simulate the barbed end depolymerization under tensile force, the Cα atoms of residues L105 and V152 of the barbed end actin subunits were pulled while the Cα atoms of residues W86 and I192 of the two pointed end actin subunits were constrained (see Supplemental Methods for details).

## Additional Information

**How to cite this article**: Lee, C.- *et al*. Regulation of actin catch-slip bonds with a RhoA-formin module. *Sci. Rep.* **6**, 35058; doi: 10.1038/srep35058 (2016).

## Supplementary Material

Supplementary Information

## Figures and Tables

**Table 1:** Evidence supporting the biological significance of actin catch-slip bonds.

		Factors perturbed	
		RhoA-formin module	Force-induced formation of K113-E195 ionic bond
Phenotypes observed	Actin catch/slip bonds phenotype	Switch from catch-slip bonds to slip-only bonds by a RhoA-formin module.	Either single mutant K113E or E195K suppresses actin catch-slip bonds; K113E/E195K double mutations allowing K-E interaction in the reversed orientation restores actin catch-slip bonds.
	Live cell phenotype	Switch of the force-mediated actin cytoskeleton alignment according to the activity of RhoA and formin^20^.	1. Yeast cells carrying either single mutant K113E or E195K exhibits growth defects (slower growth rate, abnormal cytoskeleton morphology, abnormal mitochondrial morphology). K113/E195K double mutants significantly rescue these growth defects^27^.
			2. K113E mutation in α-skeletal muscle causes nemaline myopathy^31^.
