# Supplementary material for: Regulation of actin catch-slip bonds with a RhoA-formin module
Source: Sci Rep. 2016 Oct 12;6:35058. doi: 10.1038/srep35058 (PMC5059732; doi:10.1038/srep35058)
Supplement: Supplementary Information [file srep35058-s1.doc]

**Regulation of actin catch-slip bonds with a RhoA-formin module**

Cho-yin Lee^1,2,3^, Jizhong Lou^4^, Kuo-Kuang Wen^5^, Melissa McKane^5^, Suzanne G Eskin^1,2^, Peter A. Rubenstein^5^, Shu Chien^6^, Shoichiro Ono^7^, Cheng Zhu^1,2†^, Larry V. McIntire^1,2†^

^1^Wallace H Coulter Department of Biomedical Engineering, ^2^Institute for Bioengineering and Biosciences, Georgia Institute of Technology and Emory University, Atlanta, GA,

^3^Division of Radiation Oncology, Department of Oncology, National Taiwan University Hospital and National Taiwan University Cancer Center, Taipei, Taiwan, ^4^Key Laboratory of RNA Biology, Institute of Biophysics, Chinese Academy of Sciences, Beijing, China, ^5^Department of Biochemistry, University of Iowa, Iowa City, IA, ^6^Department of Bioengineering and Institute of Engineering in Medicine, University of California at San Diego, La Jolla, CA, ^7^ Department of Pathology, Emory University, Atlanta, GA

Major Classification: Biological Sciences

Minor Classifications: Biophysics and Computational Biology; Cell Biology

^†^To whom correspondence should be addressed.

E-mail: [larry.mcintire@bme.gatech.edu](mailto:larry.mcintire@bme.gatech.edu), Tel: 1-404-894-5057, Fax: 1-404-385-5028

or E-mail: [cheng.zhu@bme.gatech.edu](mailto:cheng.zhu@bme.gatech.edu), Tel: 1-404-894-3269, Fax: 1-404-385-8109

**Supplemental Materials**

**Supplemental Methods**

**Supplemental Figures S1-S10**

**Supplemental References**

**Supplemental Methods**

**AFM protocol and parameters (adapted from the companion paper^1^ with modification)**

Our custom-made AFM, its functionalization, and the force-clamped experimental procedures for measuring lifetimes of single actin bonds have been previously described^1,2^. For G-actin/G-actin interactions (Fig. S1, *left*), the cantilever tip and the polystyrene dish surface were incubated with 2 mg/ml biotinylated BSA (Sigma Aldrich) at 4­ ^o^C overnight, washed 3 times with PBS, and incubated with 1 mg/ml streptavidin (Sigma Aldrich) for 1 hr at room temperature. After being washed 3 times with G-buffer (5 mM Tris-HCl pH 8.0, 0.2 mM CaCl_2_, 0.2 mM ATP, 0.5 mM DTT), they were incubated at 4 ^o^C for 1 hr with 1 μM biotinylated G-actin in G-buffer containing 0.00025% biotin to achieve low G-actin coating density required for single-bond measurements. The biotinylated rabbit skeletal G-actin from Cytoskeleton (Denver, CO) and yeast actin was reconstituted into G-buffer, followed by centrifugation 100,000 g for 30 min at 4­ ^o^C to remove F-actin fragments. For G-actin/F-actin interactions (Fig. S1, *right*), the cantilever tip was functionalized as for G-actin/G-actin interactions, but the polystyrene surface was functionalized with sonicated F-actin instead of actin monomer. To prepare the sonicated F-actin, 4 μM of G-actin (biotinylated actin: non-modified actin = 1:20 unless otherwise stated) was incubated in F-buffer (G-buffer + 50 mM KCl, 2 mM MgCl_2_, 1 mM ATP) for 15 min at room temperature, then sonicated for 1 min (5 seconds on, 5 seconds off), followed by 2 more cycles of 15-min incubation + 1-min sonication (Branson 1510 from Branson, Danbury, CT). The F-actin was immediately applied to the biotin/streptavidin-treated polystyrene surface with 0.00025% biotin after the last sonication. Interactions between actins were measured with F-buffer containing 0.00025% biotin to block any non-saturated binding-sites of streptavidin. The AFM was functionalized with an appropriate actin density to maintain a low binding frequency of 15-20% (Figs. S2, S4) to ensure ≥ 89% probability of forming single bonds as predicted by Poisson statistics^3^. Only single-step dissociations were analyzed, to ensure the lifetimes measured were time-to-dissociation of single bonds.

Soft cantilevers (Model: OBL-10, MLCT, Vecco, Plainview, NY) with spring constants ranging from 2-20 pN/nm were used, which were measured during each experiment using thermal fluctuations analysis^4^. A LABVIEW (National Instruments, Austin, TX) force-clamp program with feedback control was used to run the whole module for bond lifetimes measurement. The force trace data were 10-point average of the photodiode readout sampled at 2000 Hz. During each measurement cycle, the functionalized cantilever tip was driven into contact with the functionalized polystyrene surface at a speed of 200 nm/s, retracted to a short distance (10-20 nm for G-actin/G-actin interactions and 20-35 nm for G-actin/F-actin interactions) above the surface, held for 0.5 s to allow the bond formation, and retracted further at the same speed. Once a binding event was detected during the retraction, the program clamped the tether force at the preset level to measure the bond lifetime under that force.

Molecular Dynamics Simulations

MD simulations were performed with the NAMD software package^5^, under CHARM22 all-atom force field^6^ with CMAP correction. The modeled structure of the formin/actin complex containing four actin subunits and two formin FH2 domains was constructed from the crystal structure of the yeast Bni1p formin FH2 domain in complex with rabbit actins (PDB code 1Y64)^7^. It was soaked in a water box of 256×160×160Å^3^ with 626,352 atoms, including Na^+^ and Cl^-^ ions with a concentration of about 150 mM to neutralize the system. The system was subjected to sequential energy minimization steps with heavy atoms fixed, protein heavy atoms fixed, protein backbone atoms fixed, protein Cα atoms fixed and all atoms free. A 12-Å cut-off was used for van der Waals interactions, and Particle Mesh Ewald summation was used for electrostatic interaction calculation. The temperature of the systems was controlled at 310K with Langevin dynamics and the pressure was controlled at 1 atm using Nose-Hoover Langevin piston method. The energy minimized system was equilibrated for 60 ns and the resulting final snapshot was used as the initial conformation for SMD simulations. In SMD, the Cα atoms of residues L105 and V152 of the barbed end actin subunits were pulled with a constant velocity of 5 Å/ns and a spring constant of 70 pN/nm, while the Cα atoms of residues W86 and I192 of the two pointed end actin subunits were constrained. Each SMD simulation requires about 20 ns. The simulated trajectories were analyzed with Visual Molecular Dynamics^8^.

**Supplemental Figures**

**Figure S1**

**
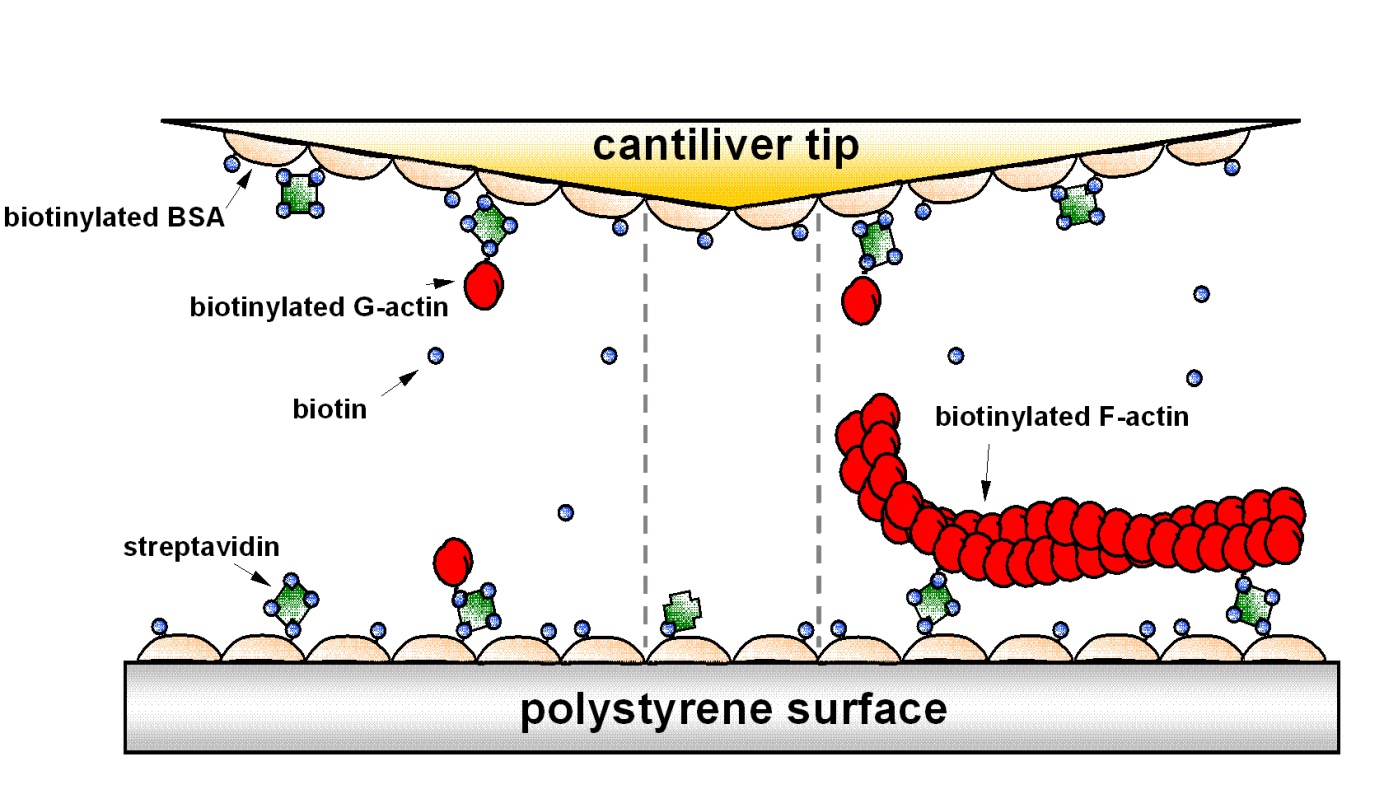
**

**Figure S1 AFM experiment.** AFM cantilever tip and polystyrene surface functionalized for G-actin/G-actin (*left*), biotin/streptavidin (*middle*) or G-actin/F-actin (*right*) interactions. Actin (red), biotin (blue), streptavidin (green), and BSA (beige) are depicted. (adapted from the companion paper^1^ with permission)

**Figure S2**

**
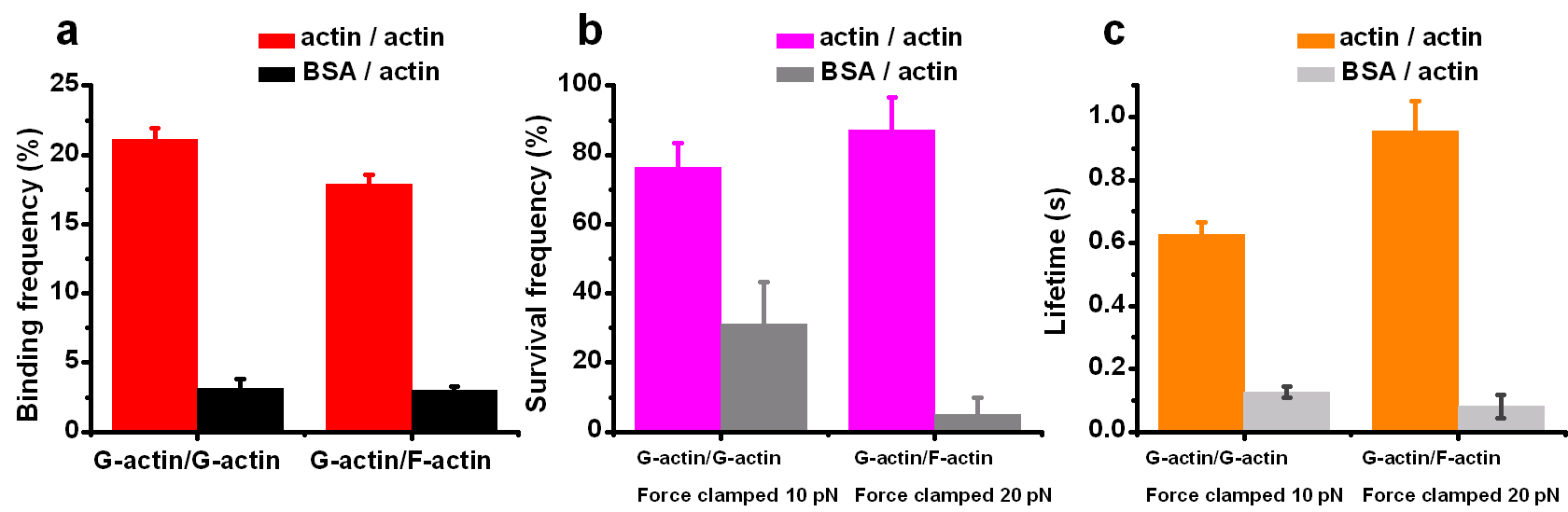
**

**Figure S2. Estimating the fraction of nonspecific binding in lifetime measurements from the binding frequency and the survival frequency.** (a) Binding frequencies of G-actin/G-actin, G-actin/F-actin, BSA/G-actin and BSA/F-actin interactions were 21.1, 18.0, 3.1 and 3 %, respectively (data presented in cited reference^1^). (b) The survival frequency is the fraction of binding that survive to the clamped tensile force and contribute to lifetime measurements. Survival frequencies of G-actin/G-actin, G-actin/F-actin, BSA/G-actin and BSA/F-actin interactions were 76.3 %, 87.2 %, 31.3 % and 5 %, respectively. These together provide the estimation of fractions of non-specific bindings in measured lifetimes of G-actin/G-actin and G-actin/F-actin interactions to be 6 % and 1 %, respectively. Data are presented as mean ± s.e.m. of 5-30 binding frequencies for each condition. Each binding frequency was estimated from the number of binding events in 50-200 contacts. (c) Bond lifetimes at optimal forces characteristic of catch bonds (10 pN and 20 pN for G-actin/G-actin and G-actin/F-actin interactions, respectively) were significantly shorter in non-specific interactions than in actin subunits interactions. Each point represents the mean ± s.e.m. of >10 measurements.

**Figure S3**


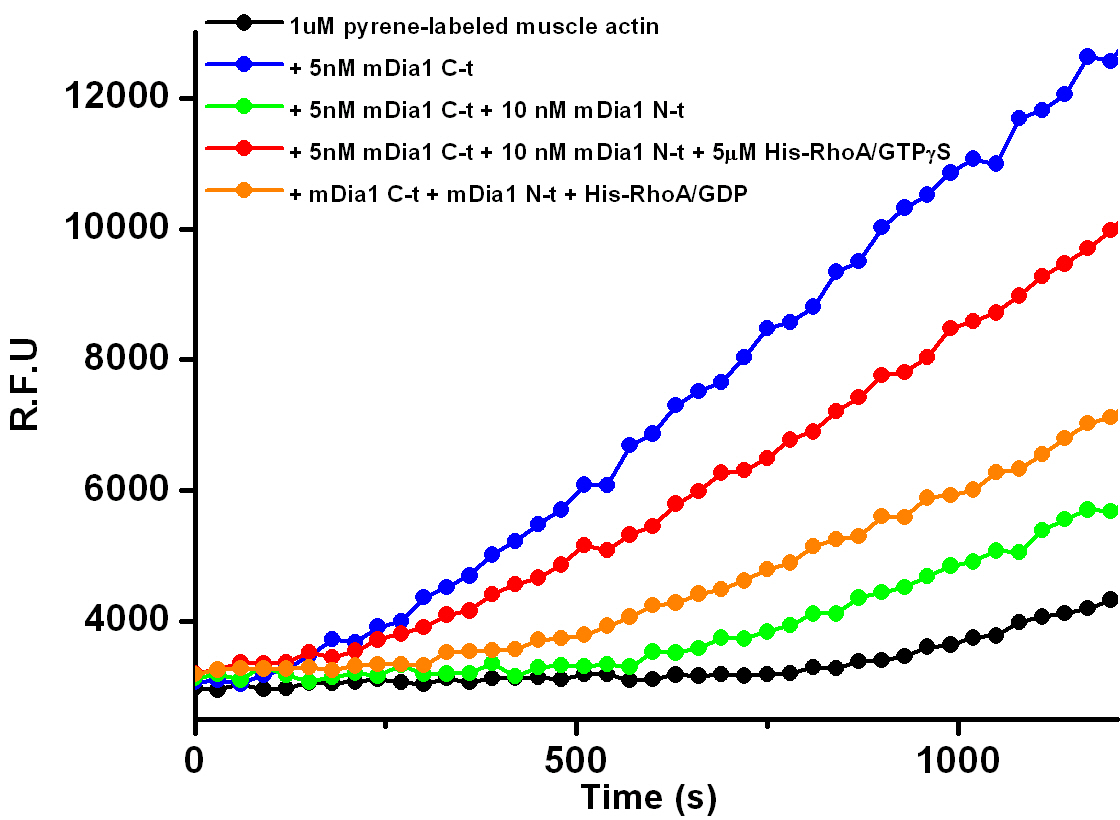


**Figure S3. Pyrene-actin polymerization assay.** 1 μM pyrene-labeled rabbit skeletal muscle actin was polymerized with mDia1 C-t, mDia N-t, and/or His-RhoA in different nucleotide states, at indicated concentrations, as previously described^9^. The increase in pyrene fluorescence indicates actin polymerization. The buffer used for the assay was the same as that for AFM experiments in this study. Actin polymerization was enhanced by mDia1 C-t (blue), which was auto-inhibited by mDia1 N-t (green). RhoA relieved the auto-inhibitory of mDia1 N-t on mDia1 C-t but the relieving effect was less potent for GDP-loaded His-RhoA than His-RhoA loaded with GTPγS (Red and Orange).

**Figure S4**


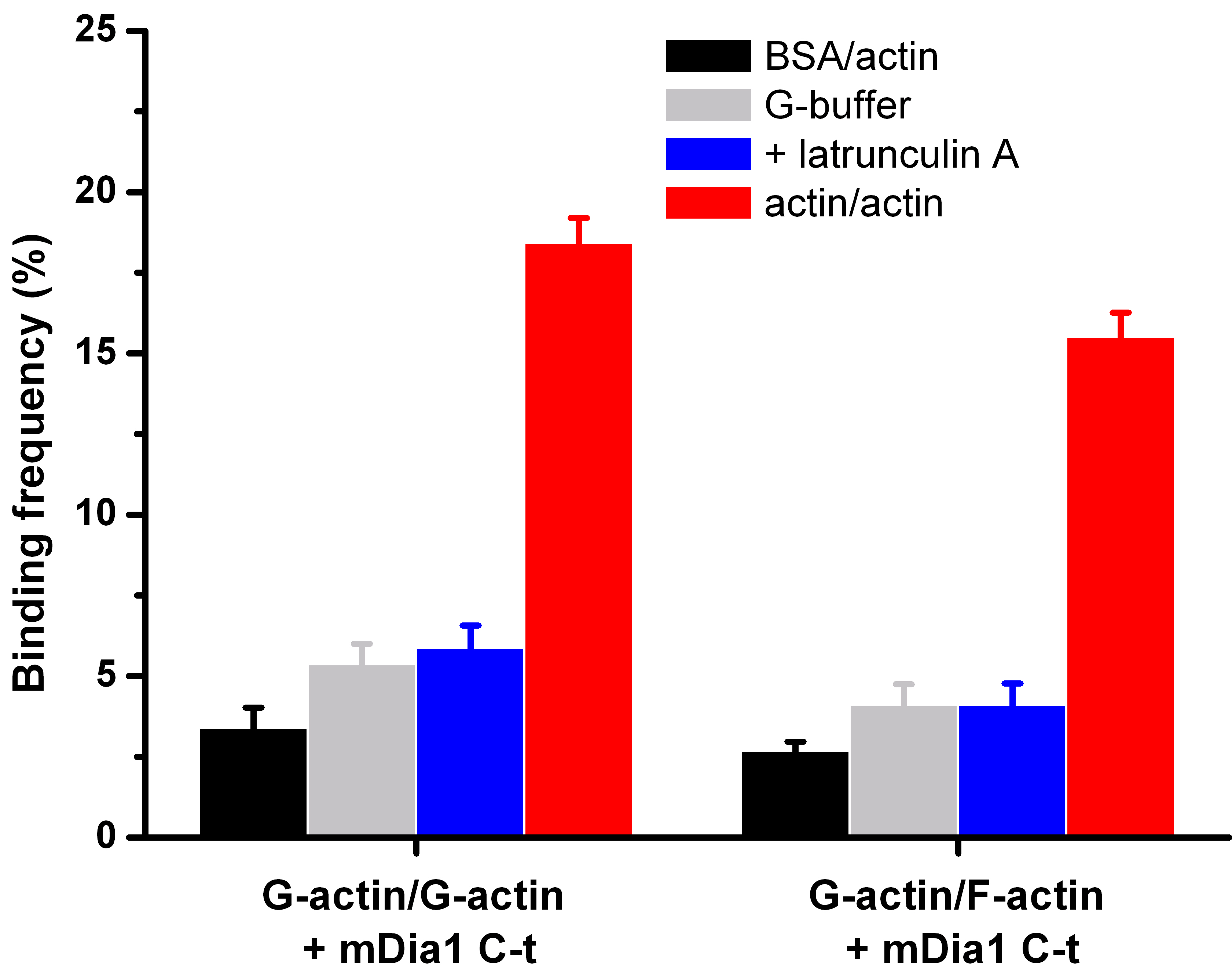


**Figure S4. Lifetimes measured in the presence of mDia1 C-t were mediated by specific actin/actin interactions.** The binding frequency of G-actin/G-actin (left) and G-actin/F-actin (right) interactions involving mDia1 C-t were significantly (p<0.001) higher than that of different control conditions in which interactions between G-actins cannot occur (by coating the cantilever tip with BSA instead of G-actin, by using G-buffer instead of F-buffer as the working buffer, or by adding 2 μM latrunculin A). Data are presented as mean ± s.e.m. of 10-30 binding frequencies for each condition. Each binding frequency was estimated from the number of binding events in 100-200 contacts.

**Figure S5**


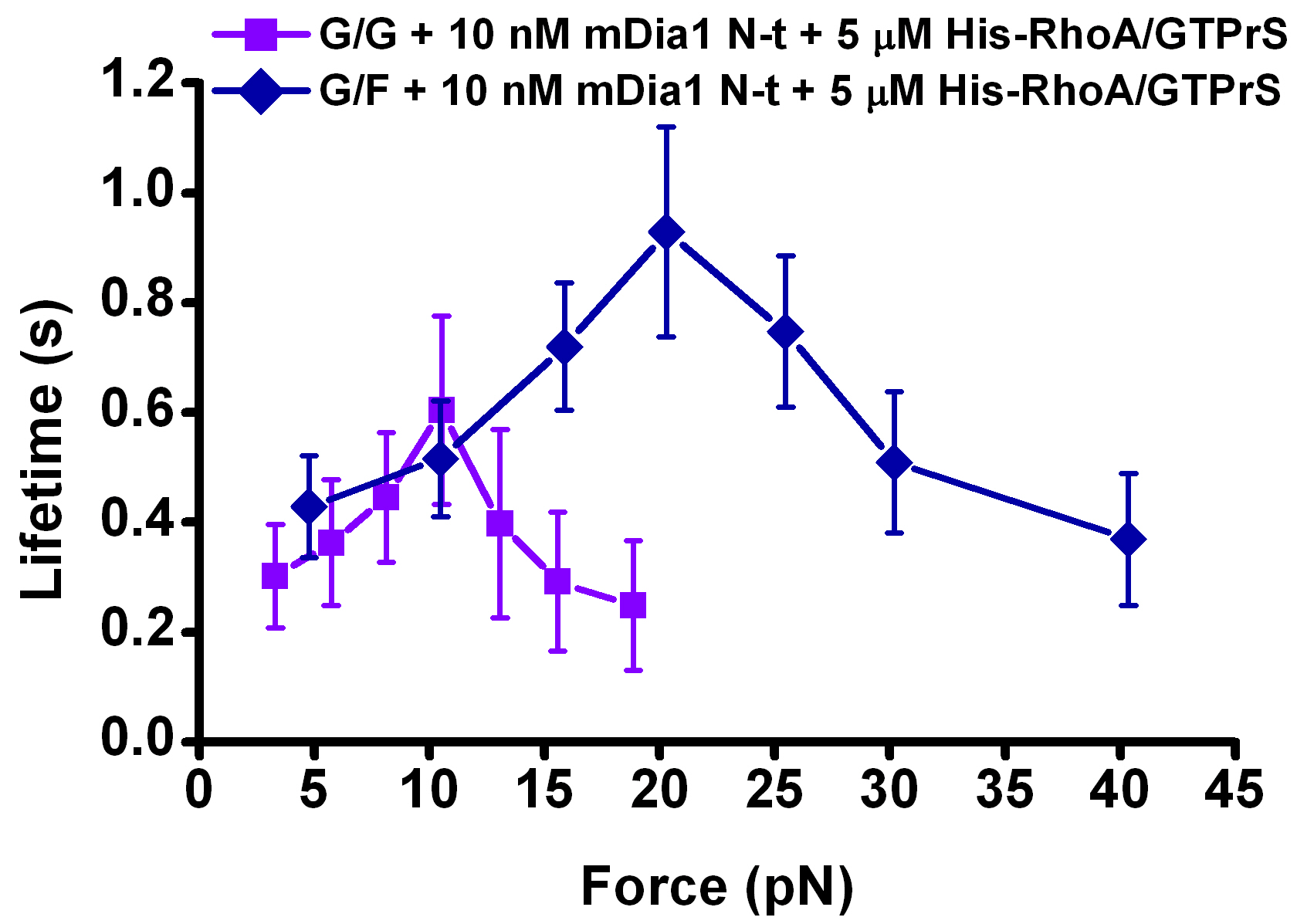


**Figure S5. His-RhoA and mDia1 N-t together had no effect on actin catch-slip bonds.** (compared with Fig. 2) The observed relieving effect of RhoA was specific and depended on rescuing the activity of mDia1 C-t from mDia1 N-t/mDia1 C-t auto-inhibition. Each point represents the mean ± 95% C.I. of >30 measurements.

**Figure S6**


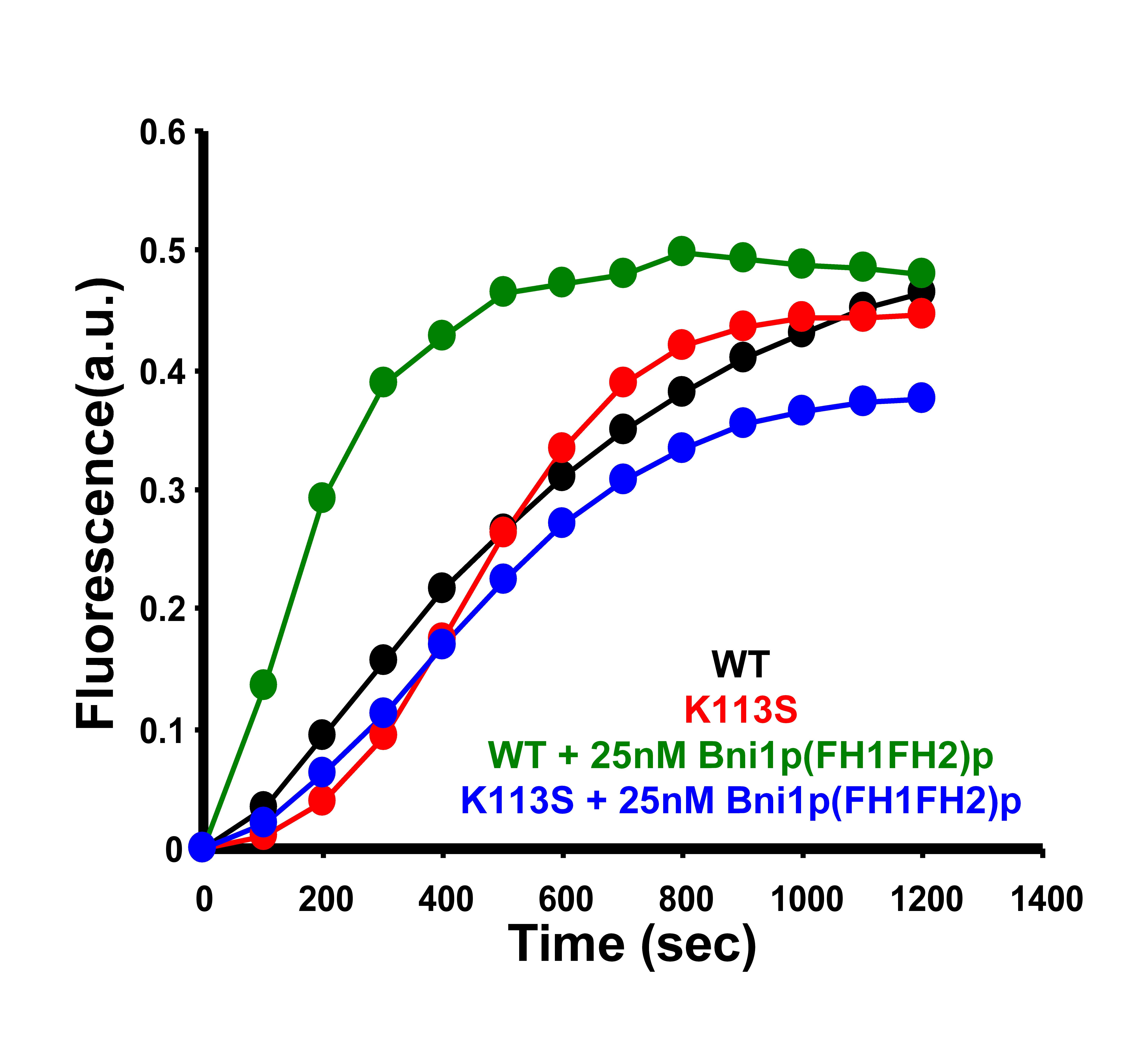


**Figure S6. The effect of Bni1 on pyrene-actin polymerization assay was suppressed by K113S mutant.** 2 μM wild-type (WT) or mutant (K113S) yeast actin containing 5% pyrene-labeled WT actin was polymerized with or without 25 nM Bni1p(FH1-FH2)p. The increase in pyrene-fluorescence indicates actin polymerization. The effect of Bni1p(FH1-FH2)p on pyrene-actin polymerization assay was suppressed by K113S mutant.

**Figure S7**

**
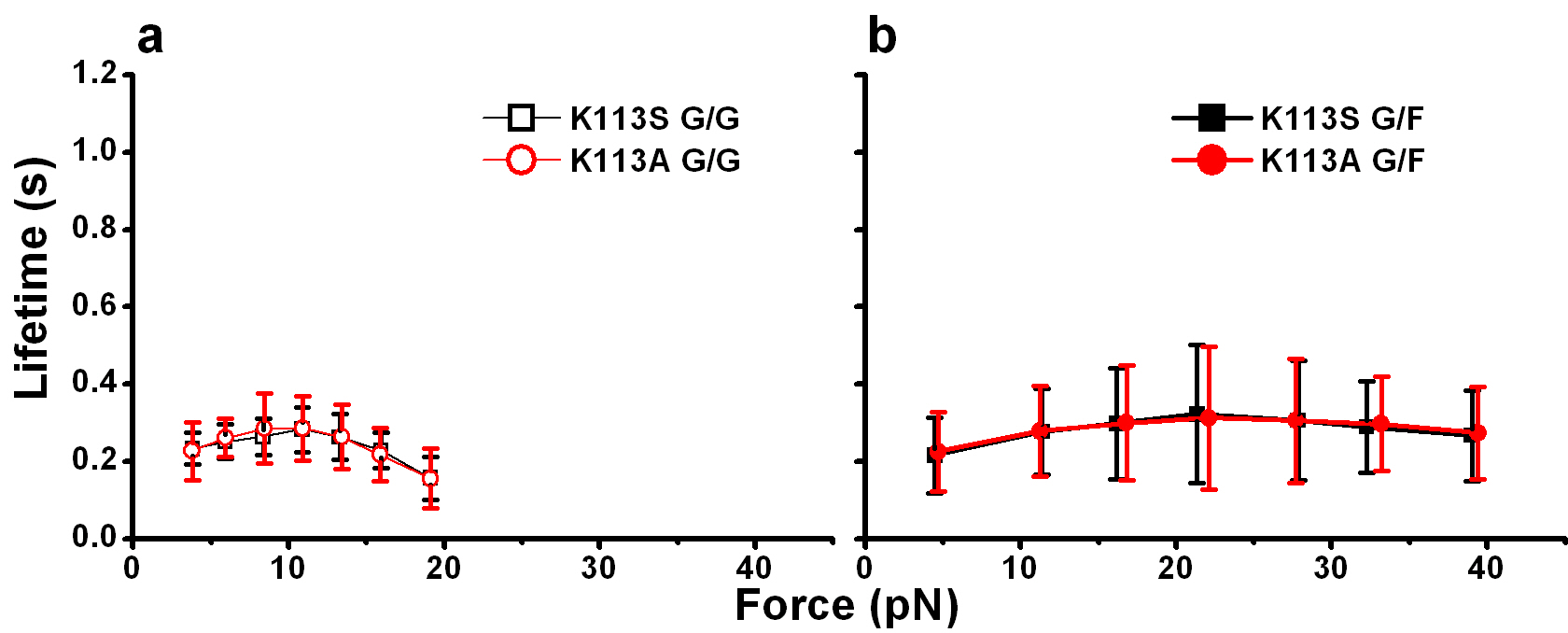
**

**Figure S7. The effect of yeast actin mutations K113S or K113A on actin catch-slip bonds.** (a) G-actin/G-actin catch-slip bonds were suppressed by yeast actin mutation K113S(open black square, data presented in^1^) or K113A(open red circle)(compared with Fig 3a). (b) G-actin/F-actin catch-slip bonds were suppressed by K113S(black square) or K113A(red circle)(compared with Fig. 3e). Each point represents the mean ± 95% C.I. of >30 measurements.

**Figure S8**

**
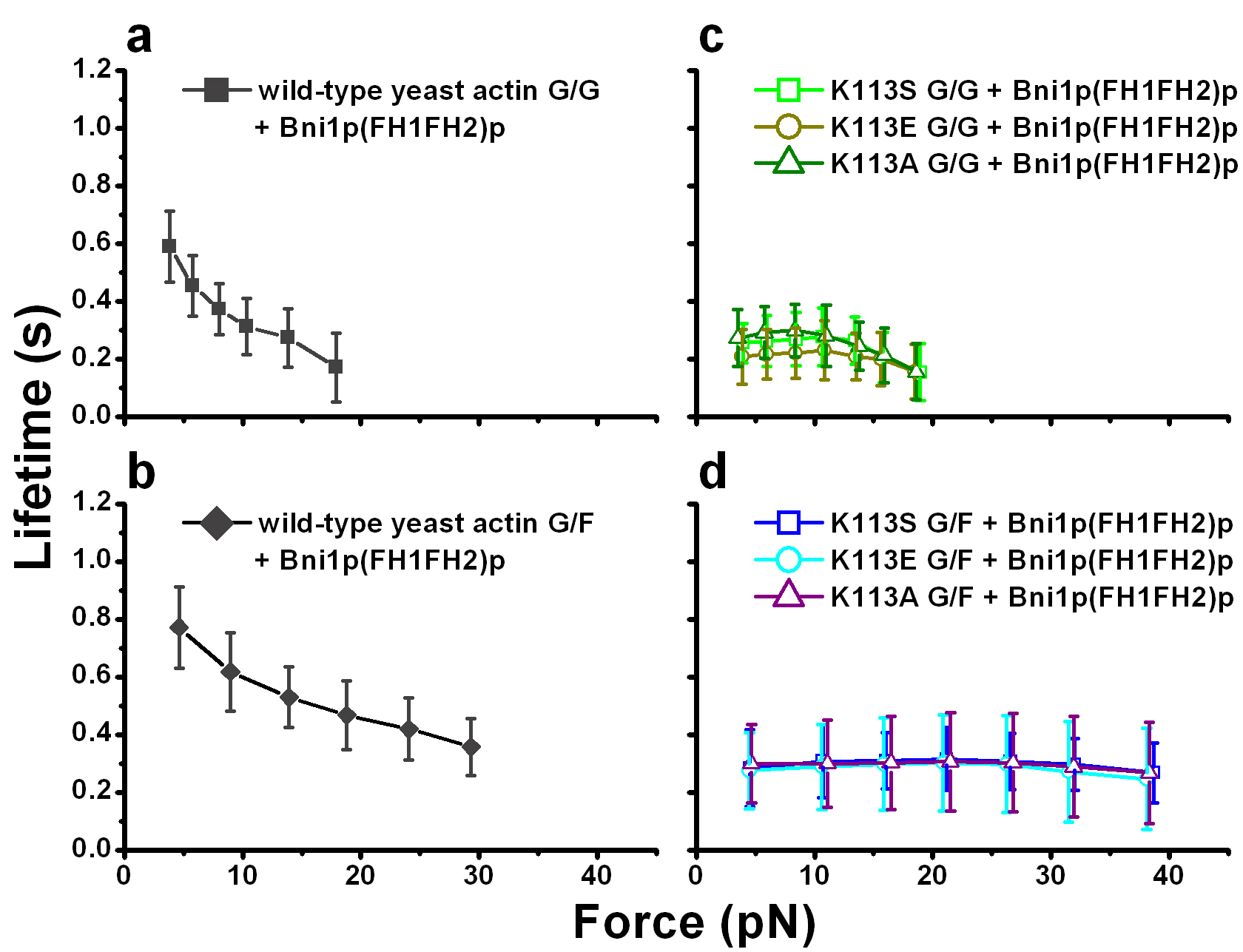
**

**Figure S8. Formin regulation of actin catch-slip bonds was abolished by actin mutant K113S.** Catch-slip bonds of wild-type yeast actin in (a)G-actin/G-actin and (b)G-actin/F-actin interactions were converted to slip bonds by 25 nM Bni1p(FH1-FH2)p. (c and d) Mutations on yeast actin K113 residues, K113S(open square), K113E(open circle) and K113A(open triangle), abolished the effect of Bni1p(FH1-FH2)p on G-actin/G-actin(c) and G-actin/F-actin(d) interactions. Each point represents the mean ± 95% C.I. of >30 measurements.

**Figure S9**

**
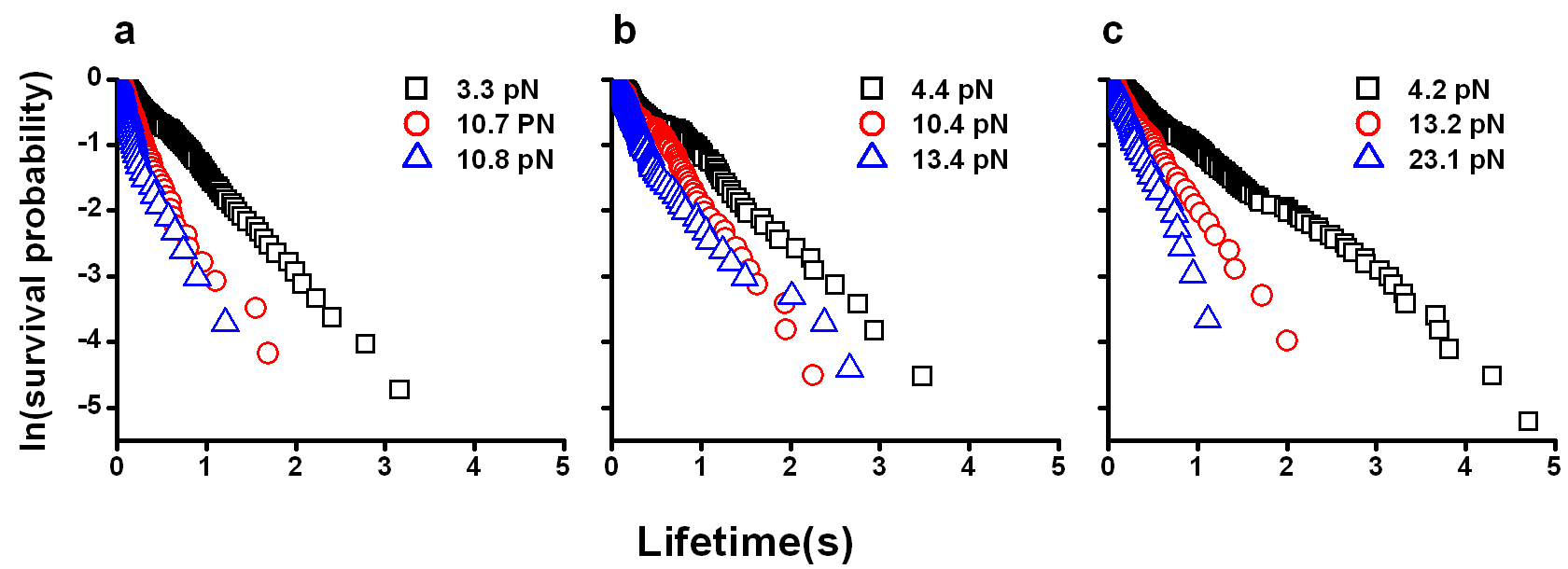
**

**Figure S9. Survival frequency versus life time.** Single exponential lifetime distributions exemplified by the linear semilog plots of survival frequency(i.e., fraction of bonds survived longer than a given time) versus lifetime for the G-actin/G-actin interaction with 5 nM mDia1 C-t (a), the G-actin/F-actin interaction with 5 nM mDia1 C-t (b), the G-actin/F-actin interaction with 5nM mDia1 C-t and 2μM Tmod3 (c), at representative force bins presented in Figure 2a and Figure 2d.

**Figure S10**

**
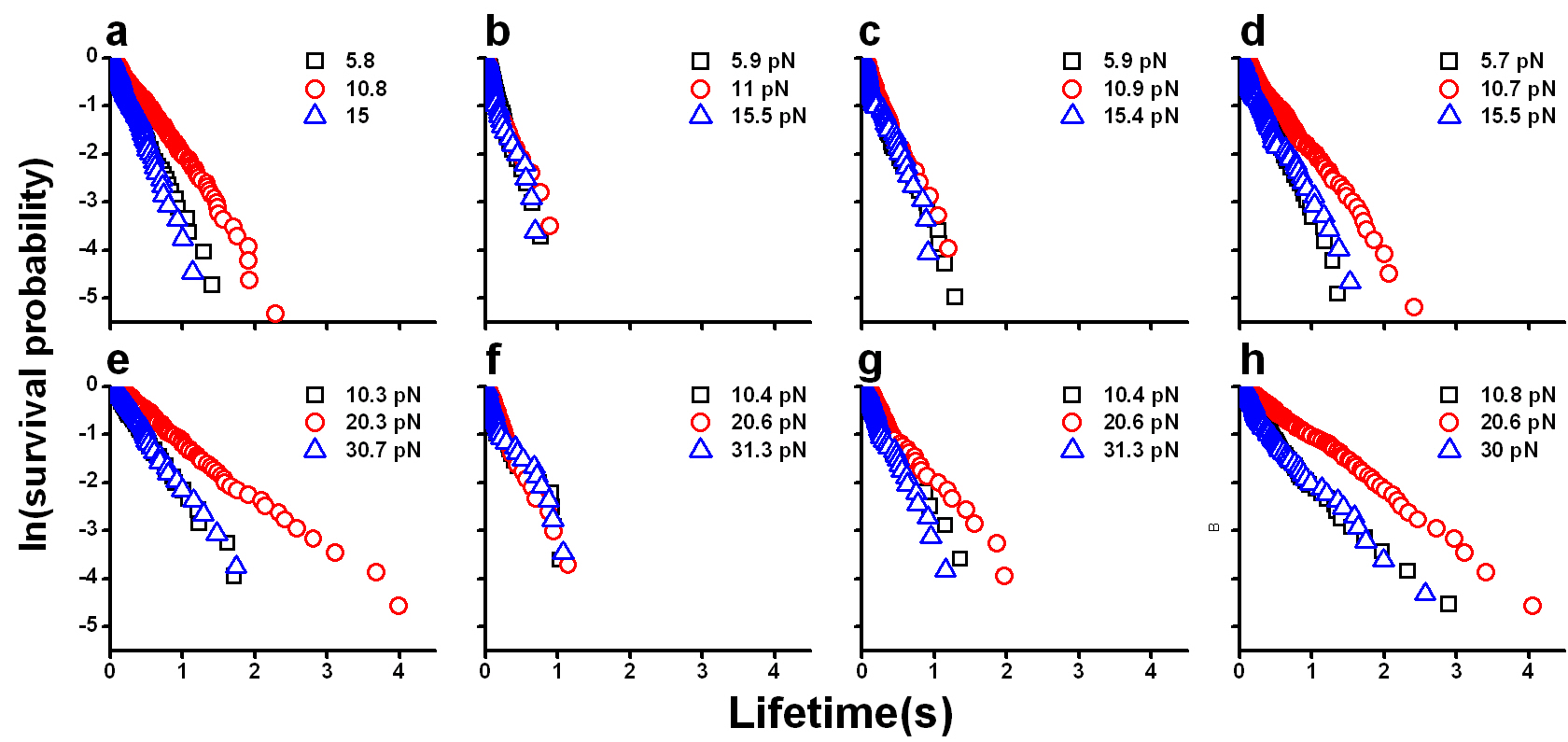
**

**Figure S10. Survival frequency versus life time.** (a-h) Single exponential lifetime distributions exemplified by the linear semilog plots of survival frequency (i.e., fraction of bonds survived longer than a given time) versus lifetime at every other force bin presented in Figure 4a-4h, respectively. (a) wild type yeast actin, G-actin/G-actin interaction; (b) K113E mutant, G-actin/G-actin interaction; (c) E195K mutant, G-actin/G-actin interaction; (d) E/K reverse double mutant, G-actin/G-actin interaction; (e) wild type yeast actin, G-actin/F-actin interaction; (f) K113E mutant, G-actin/F-actin interaction; (g) E195K mutant, G-actin/F-actin interaction; (h) E/K reverse double mutant, G-actin/F-actin interaction.

**Supplemental References**

^1^ Lee, C. Y. et al., Actin depolymerization under force is governed by lysine 113:glutamic acid 195-mediated catch-slip bonds. *Proc Natl Acad Sci U S A* **110** (13), 5022 (2013).

^2^ Kong, F. et al., Demonstration of catch bonds between an integrin and its ligand. *J. Cell Biol.* **185** (7), 1275 (2009); Yago, T. et al., Platelet glycoprotein Ibalpha forms catch bonds with human WT vWF but not with type 2B von Willebrand disease vWF. *J Clin Invest* **118** (9), 3195 (2008); Marshall, B. T. et al., Direct observation of catch bonds involving cell-adhesion molecules. *Nature* **423** (6936), 190 (2003).

^3^ Zhu, C., Long, M., Chesla, S. E., and Bongrand, P., Measuring receptor/ligand interaction at the single-bond level: experimental and interpretative issues. *Ann Biomed Eng* **30** (3), 305 (2002).

^4^ Wu, J., Fang, Y., Yang, D., and Zhu, C., Thermo-mechanical responses of a surface-coupled AFM cantilever. *J Biomech Eng* **127** (7), 1208 (2005).

^5^ Phillips, James C. et al., Scalable Molecular Dynamics with NAMD. *J. Comput. Chem.* **26**, 1781 (2005).

^6^ MacKerell, A., Jr. et al., All-atom empirical potential for molecular modeling and dynamics Studies of proteins. *Journal of Physical Chemistry. B* **102**, 3586 (1998).

^7^ Otomo, T. et al., Structural basis of actin filament nucleation and processive capping by a formin homology 2 domain. *Nature* **433** (7025), 488 (2005).

^8^ Humphrey, William, Dalke, Andrew, and Schulten, Klaus, VMD: Visual molecular dynamics. *Journal of Molecular Graphics* **14**, 33 (1996).

^9^ Li, F. and Higgs, H. N., The mouse Formin mDia1 is a potent actin nucleation factor regulated by autoinhibition. *Curr. Biol.* **13** (15), 1335 (2003); Li, F. and Higgs, H. N., Dissecting requirements for auto-inhibition of actin nucleation by the formin, mDia1. *J Biol Chem* **280** (8), 6986 (2005).
